# Alternatives to currently used antimalarial drugs: in search of a magic bullet

**DOI:** 10.1186/s40249-016-0196-8

**Published:** 2016-11-04

**Authors:** Akshaya Srikanth Bhagavathula, Asim Ahmed Elnour, Abdulla Shehab

**Affiliations:** 1 Department of Clinical Pharmacy, University of Gondar-College of Medicine and Health Sciences, School of Pharmacy, Gondar, Ethiopia; 2 Pharmacy College, Fatima College of Health Sciences, Al Ain, Abu Dhabi United Arab Emirates; 3 Department of Internal medicine, College of Medicine and Health Sciences, UAE University, Al Ain, Abu Dhabi United Arab Emirates

**Keywords:** Malaria, Drug development, Medicine for malaria venture, Chemotherapy, Tafenoquine, Fosmidomycin, Novel antimalarial drugs, Artemisinin derivatives

## Abstract

Malaria is a major cause of morbidity and mortality in many African countries and parts of Asia and South America. Novel approaches to combating the disease have emerged in recent years and several drug candidates are now being tested clinically. However, it is long before these novel drugs can hit the market, especially due to a scarcity of safety and efficacy data.

To reduce the malaria burden, the Medicines for Malaria Venture (MMV) was established in 1999 to develop novel medicines through industry and academic partners’ collaboration. However, no reviews were focused following various preclinical and clinical studies published since the MMV initiation (2000) to till date.

We identify promising approaches in the global portfolio of antimalarial medicines, and highlight challenges and patient specific concerns of these novel molecules. We discuss different clinical studies focusing on the evaluation of novel drugs against malaria in different human trials over the past five years.

The drugs KAE609 and DDD107498 are still being evaluated in Phase I trials and preclinical developmental studies. Both the safety and efficacy of novel compounds such as KAF156 and DSM265 need to be assessed further, especially for use in pregnant women. Synthetic non-artemisinin ozonides such as OZ277 raised concerns in terms of its insufficient efficacy against high parasitic loads. Aminoquinoline-based scaffolds such as ferroquine are promising but should be combined with good partner drugs for enhanced efficacy. AQ-13 induced electrocardiac events, which led to prolonged QTc intervals. Tafenoquine, the only new anti-relapse scaffold for patients with a glucose-6-phosphate dehydrogenase deficiency, has raised significant concerns due to its hemolytic activity. Other compounds, including methylene blue (potential transmission blocker) and fosmidomycin (DXP reductoisomerase inhibitor), are available but cannot be used in children.

At this stage, we are unable to identify a single magic bullet against malaria. Future studies should focus on effective single-dose molecules that can act against all stages of malaria in order to prevent transmission. Newer medicines have also raised concerns in terms of efficacy and safety. Overall, more evidence is needed to effectively reduce the current malaria burden. Treatment strategies that target the blood stage with transmission-blocking properties are needed to prevent future drug resistance.

**Electronic supplementary material:**

The online version of this article (doi:10.1186/s40249-016-0196-8) contains supplementary material, which is available to authorized users.

## Multilingual abstract

Please see Additional file 1 for translations of the abstract into the five offical working languages of the United Nations.

## Background

Malaria is an infectious disease caused by the protozoa of the genus *Plasmodium*, transmitted through the bite of the female *Anopheles* mosquito. It is a major public health problem in many endemic countries including Sub-Saharan Africa (SSA); in 2015, an estimated 438 000 malaria deaths were reported globally [1]. Human pathogenic *Plasmodium* species include *Plasmodium falciparum*, *P. vivax*, *P. ovale*, *P. malariae*, and *P. knowlesi*. Sporozoites are injected into the skin through a mosquito bite, invading hepatocytes and causing liver infection. The merozoites released from the liver rapidly infect the erythrocytes during the erythrocytic stage. Multiple rounds of the erythrocytic stage produce larger numbers of parasites that invade the blood, consequently causing clinical illness. Erythrocytic parasites develop into sexual gametocytes and are transmitted to humans through a mosquito bite.

The *Plasmodium*-infected female *Anopheles* mosquito is the deadliest known disease vector causing as many deaths as deaths from HIV/AIDS and tuberculosis [2]. In 2008, the World Health Organization (WHO) initiated the Global Malaria Action Plan (GMAP) to reduce rates of malaria incidence and mortality by at least ten-fold by 2030. Despite numerous advances over the past decade, new drugs are urgently needed. To reduce the malaria burden in developing countries, the not-for-profit organization Medicines for Malaria Venture (MMV) was established in 1999. Its main goal is to initiate collaborations with industry and academic partners in order to develop novel approaches to combat malaria [3]. This has led to the design and discovery of new medicines for human malaria cases. Between 2000 and 2015, malaria incidence rates decreased globally by 37 % and mortality rates decreased by 60 %. Most malaria cases (89 %) and deaths (91 %) from malaria globally were reported in SSA [4]. The emergence of resistance to traditional therapies including chloroquine, primaquine, quinine, and mefloquine has also revealed novel antimalarial targets. In the past decades, a consortium of researchers from academia and industry was created to develop new remedies focusing on chemotypes [5]. Consequently, the discovery of artemisinin-based combination therapies (ACTs) by Chinese scientists has tremendously benefitted hundreds of thousands of patients. However, resistance to antimalarial drugs continues to pose a major threat to malaria eradication [6]. Furthermore, recent advances in the development of species-specific malaria vaccines have emerged as the most prominent approach to eradicating malaria.

24 malarial vaccines are currently being tested in 99 clinical sites in Africa and 30 in Southeast Asia [7]. The most advanced recombinant protein-based malaria vaccine is Mosquirix™ (RTS, S), a combination of 25 % fusion protein RTS and 75 % wild-type hepatitis B surface antigen (HBsAg). The vaccine is effective against *P. falciparum* malaria and was developed by GlaxoSmithKline (GSK), the PATH Malaria Vaccine Initiative, and other partners. In a late-stage Phase III trial, Mosquirix™ showed poor efficacy with only 27 % protection against severe malaria in infants [8]. The European Medicines Agency approved the use of Mosquirix™ in young African children in July 2015, although final consent from the WHO is still needed. While these vaccines may provide partial protection, their widespread use in Africa may be affected by high costs, as well as compliance and feasibility challenges.

### Challenges for the eradication of malaria

Since 2000, malaria infection rates have been reduced by over 50 %, but complete eradication is still a challenge [9]. To completely wipe out malaria, multifaceted strategies are essential. One approach is single exposure radical cure and prophylaxis (SERCaP), proposed as a global agenda in 2007 for identifying ideal drugs to treat malaria [10]. International guidelines have recommended the use of ACTs as a first-line treatment on a three-day course schedule, as ACTs, particularly artesunate, can rapidly reduce the parasitic load by at least 10 000-fold within 48 h of the parasitic life cycle, resulting in >95 % clearance of initial infection. However, subtherapeutic doses and non-adherence are additional barriers promoting the emergence of resistant malarial strains and contributing to treatment failure. Medicines with longer durations of drug action and higher minimum inhibitory concentrations (MICs) in the plasma for at least one week can suppress the parasitic load and offer post-exposure and post-treatment protection. Chemoprotectants are an emerging class of drugs. Sulfadoxine-pyrimethamine is a chemoprotectant combination drug that was widely used among patients until the emergence of resistance last decade [11, 12]. Novel chemoprotective agents acting against the schizont stage of the malaria parasite are needed to prevent merozoite production from blood cells.

In this study, we reviewed various preclinical and clinical studies published during the period directly following the establishment of the Medicines for Malaria Venture (2000) and 2015.

We discuss different preclinical and clinical studies focusing on the evaluation of novel drugs against malaria in different human trials over the past five years registered in the clinicaltrials.gov database [13] (see Table 1). We also address additional approaches to treating malaria with a special focus on safety.

**Table 1:** Overview of some of the ongoing clinical trial performed in Malaria

Agents	Class	Target name	Clinical Trial	Control	Study population	Dosage	Reference
Phenotypic assay							
KAE609(cipargamin)*	Spiroindolone	Na + −ATPase 4	Phase II	Not specified	Adult (>18 years and older)	800 mg single dose for falciparum patients	NCT01836458
MMV390048	PfPI4K	Phosphatidylinositol-4 kinase	Phase I	Not specified	Healthy volunteers	20 mg single dose as suspension for falciparum patients	NCT02281344
KAF156*	PfCARL	Cyclic amine resistance locus inhibitor	Phase II	Not specified	Adult (>18 years)	800 mg/day for 3 days and 800 mg single dose for uncomplicated falciparum and vivax	NCT01753323
DSM265	PfDHODH	Dihydroorotate dehydrogense	Phase II	Not specified	Adults (>18 years)	400 mg single dose for uncomplicated falciparum and vivax	NCT02123290
Synthetic Molecules							
OZ277 + Piperaquine	PfATP6	Pf-encoded sarcoplasmic endoplasmic reticulum calcium ATPase	Phase II-III	Dihydroartemisinin + piperaquine	Adult (>18 years)	Not specified	NCT02461186
OZ439 + Piperaquine	endoperoxide ozonide	1,2,4-trioxolane	Phase II-III	Not specified	6 months to 70 years	OZ439 -800 mg single dose +1440 mg piperaquine for uncomplicated falciparum patients	NCT02083380
Artemisone (BAY-44-9585) + Mefloquine	Artemisin derivative		Withdrawn	Artesunate	>16 years and older	Artemisone 4 mg/kg/day for 3 days, mefloquine 15 mg/kg/day for uncomplicated falciparum malaria	NCT00936767
Aminoquinoline scaffolds							
Ferroquine	4-aminoquinolines	Ferrocene − 4-aminoquinoline	Phase II Terminated	Placebo and Artesunate	>14 years and older	Ferroquine different doses with artesunate 4 mg/kg/day, once daily for 3 days vs placebo for uncomplicated falciparum malaria	NCT00988507
AQ-13	4-aminoquinolines	Unknown	Phase II	Coartem	18 years and older	Two (350 mg) capsules on day 1 and 2. One (350 mg) capsule on day 3 for for uncomplicated falciparum malaria	NCT01614964
Tafenoquine	8-aminoquinolines	Unknown	Phase III	Placebo, chloroquine, primaquine	16 years and older	Tafenoquine 150 mg vs chloroquine different doses vs placebo for plasmodium vivax malaria	NCT02216123
Antibiotics							
Fosmidomycin	Streptomycin lavenduale	DOXP pathway	Phase IIa	unspecified	1 year to 60 years	Fosmidomycin 450 mg twice daily + piperaquine 320 mg once daily for 3 days for uncomplicated falciparum malaria.	NCT02198807
Other agents							
Methylene blue + Primaquine	Phenothiazin dye	Unknown	Phase I	Healthy volunteers	18 to 60 years	Primaquine 45 mg + methylene blue 600 mg single dose compared in patients with Normal G6PD vs G6PD deficiency	NCT01668433

Source: clinicaltrial.org, *Completed: waiting for results

## Methods

To summarize the existing evidence related to the portfolio of novel antimalarial drugs, we conducted a systematic review using the Preferred Reporting Items for Systematic Reviews and Meta-Analyses (PRISMA) Statement [14]. We searched publicly available sources such as PubMed, Web of Science, clinicaltrials.gov, and drug company websites. Original and unoriginal peer-reviewed articles published between 2000 (after the establishment of MMV) and 2015 were retrieved. We included preclinical studies and all clinical trial phases. If trial results were unavailable, we referenced the study either by its clinicaltrial.gov identifier or using the company press release newsletters.

Two of the authors of this paper (ASB and AAE) checked all potentially relevant studies and reached a consensus on all items. One of the authors (ASB) screened the titles and abstracts. Two authors (ASB and AAE) selected the studies for inclusion after reviewing full-text articles. The following keywords were entered into the search field to search for titles, abstracts, and index terms: novel* AND antimalarial* AND preclinical* AND clinical trials* AND plasmodium falciparum* AND plasmodium vivax* AND malaria* AND medicine for malaria venture*. Data were updated in January 2016.

Extracted data were based on information reported in or calculated from the included studies. Authors were not contacted for additional information regarding the drug or trial information. The following information was retrieved from clinicaltrials.gov therapeutic agent, class of drug, site of action, clinical phase, control group, study population, and therapeutic doses. We only examined information on antimalarial drug efficacy and safety.

## Pharmacological approaches

### Phenotypic molecules for malaria

#### KAE609

Advances in automation and phenotypic assay screening techniques have aided the discovery of innovative compounds effective against both asexual and sexual stages of *P. falciparum*. Spiroindolone KAE609 (cipargamin), a potential Na^+^-ATPase 4 ion channel (PfATP4) inhibitor, was discovered by scientists from the Swiss Tropical and Public Health Institute and developed by the Novartis Institute of Tropical Diseases in Singapore. KAE609 originated from the high-throughput phenotypic screening of nearly 12 000 natural compounds evaluated for their activity against erythrocytic stages of *P. falciparum* [15]. Encouraging results were reported in a Phase I human trial with elevated MICs in the plasma for several days, and total efficacy doses of 300 mg (single) and 150 mg (multiple) for three days [16, 17]. Interestingly, KAE609 is seven times more potent than artesunate and 40 times more potent than 4-aminoquinolines [18]. Results from a recent Phase II clinical trial conducted among Thai patients indicated a clearance half-life of 0.90 h for *P. falciparum* and 0.95 h for *P. vivax*. Furthermore, the mean terminal half-life for the elimination of KAE609 was 20.8 h, supporting a once-daily oral dosing regimen [18]. The promising profile of KAE609 will be evaluated further in upcoming early phase trials. In vitro, KAE609 showed active against artemisinin-resistant K 13 mutant parasite and prevents recrudescence of dihydeoartemisinin (DHA)- arrested ring at minimal concentration (50 nM) [19]. Thus can as a broad range antimalarial and in treatment of multidrug-resistant *P.facliparum* malaria.

#### DDD107498

Advances in natural-product-based screening techniques have improved our understanding of medicinal chemistry via chemoinformatics. A high-throughput screening of more than 4 700 compounds resulted in a promising scaffold, which led to the discovery of DDD107498, a novel phenotypic molecule that specifically acts against liver-stage *P. falciparum* malaria. This molecule was developed at the University of Dundee, UK by a research consortium [20]. The DDD107498 compound is a 2,6-disubstituted quinoline-4-carboxamide scaffold effective against the liver (schizont formation) stage. In vitro assays against different *P. falciparum* laboratory strains, such as artemisinin-resistant strains, chloroquine-, amodiaquine- and mefloquine-resistant strains, revealed a low micromolar range against the parasite. In addition, the compound impaired the growth of other strains including *P. berghei* and *P. yoelii* during their schizont formation stage. DDD107498 may be effective against multidrug resistant *Plasmodium* strains (Dd2 and 7G8). Remarkably, the ex vivo efficacy of DDD107498 has been shown to be higher than artesunate against *P. falciparum* (median EC_50_ = 0.81 nM [range 0.29–3.29 nM]) and *P. vivax* (median EC_50_ = 0.51 nM [range 0.25–1.39 nM]) [20]. DDD107498 has shown excellent oral bioavailability and a longer plasma half-life, which is preferable for single-dose treatment in vitro. These results suggest that DDD107498 can achieve complete parasitic clearance in the blood stage by rapid killing for more than 48 h. DDD107498 is currently in the developmental stage and needs to be tested for approval in human clinical trials.

#### KAF156

KAF156 (also known as imidazolopiperazine), a promising chemoprevention molecule, is a cyclic amine resistance locus inhibitor (PfCARL) developed by the Novartis research consortium [21]. In vitro KAF156 is active against uncomplicated *P. falciparum* and *P. vivax* strains in the liver, asexual erythrocytic, and transmission stages. One recently published paper reported a KAF156 Phase II proof-of-concept trial [22] conducted among Vietnamese and Thai monoinfection patients, who were treated with 400 mg/day for three days and a single 800 mg dose. No efficacy data are yet available on KAF156 and no predictions can be made about its potential as a future antimalarial drug. Adverse events reported with higher doses of KAF156 include sinus bradycardia, thrombocytopenia, and hypokalemia. Further studies are needed to evaluate the molecule’s side effects.

#### DSM265

DSM265, a dihydroorotate dehydrogenase (DHODH) inhibitor acting against the liver (schizont formation) stage, is proving to be promising as a one-dose (400 mg) malaria cure in a Phase I trial in healthy volunteers, with an encouraging safety profile. DSM265 is currently in the clinical developmental stage (Phase II) in Peru (NCT02123290). Its activity against uncomplicated *P. falciparum* and *P. vivax* parasites is being assessed in adult patients using a single dose treatment (400 mg) [23]. However, no clinical data are yet available to confirm DSM265 as a potential antimalarial armory. Although DSM265 showed robust results in Phase I trials, further studies are needed to predict its safety for use in pregnant women.

In most countries in SSA, malaria in pregnancy contributes to significant maternal and perinatal mortality. It is not recommended to use ACTs during the first trimester due to side effects observed in preclinical models [24]. Currently, sulfadoxine-pyrimethamine is used in pregnant women as an intermittent preventive treatment to reduce infections and improve pregnancy outcomes. Several optional antibacterials and antifolate combinations have emerged including azithromycin-chloroquine, mefloquine, and dihydroartemisinin-piperaquine. Antibacterial combinations potentially reduce the risk of sexually transmitted diseases to mothers and newborns [25]. Studies registered in clinicaltrials.gov on co-trimoxazole prophylaxis for prevention of malaria in pregnancy (NCT01053325) and co-infection with malaria and HIV in women (NCT00970879) were completed in 2013, but results have not yet been published. Moreover, mefloquine has shown significant benefits but may cause nausea and neuropsychiatric side effects [26]. To fulfill the GMAP portfolio, determining the safety of novel chemoprotective molecules in pregnancy should be considered a priority in clinical investigations.

### Other compounds under development

Several molecules are currently being tested in preclinical models. Examples include SJ557733, developed in collaboration between St. Jude Children’s Research Hospital, TN, USA and Rutgers University, NJ, USA [27], and PA21A092 developed at Drexel University, PA, USA [28]. Both molecules target the PfATP4 of multiple *Plasmodium* species at different stages of infection. Another similar phenotypic molecule known as MMV390048, developed by researchers at the University of Cape Town in South Africa, targets lipid phosphatidyl inositol 4-kinase (PfPI4K) [29]. The MMV390048 research group has completed a Phase I trial on healthy African volunteers for the first time, but results have not yet been published (registered in clinicaltrials.gov; NCT02230579). Although additional novel phenotypic molecules are currently being clinically tested against malaria (see Table 2), more studies are needed to elucidate their clinical effectiveness and safety. Genetic polymorphism in *pfcrt* is associated with chloroquine resistance. Additional polymorphisms (*dhfr* and *dhps*) for sulfadoxine-pyrimethamine and polymorphism of *P. falciparum* multidrug resistance protein 1 (*pfmdr1*) are associated with resistance to chloroquine, mefloquine, quinine, and artemisinin [30]. Novel loci such as encoding the mu chain of the adopter protein 2 (ap2-mu), *P. falciparum* ap2-mu (Pfap2-mu) homologue [27], gene mutations encoding *pfmdr1*, and sarco-endoplasmic reticulum calcium ATPase6 (PfSERCA) [31] may be associated with antimalarial resistance. Emerging evidence shows that *pfmdr 1*, *pfcrt*, and *pf3d7-1343700 Kelch propeller (K13-propeller)* mutations are potential markers indicating that *P. falciparum* is developing resistance to artemisinin and its derivatives [32, 33].

**Table 2:** Novel antimalarial candidates in preclinical stage

References	Molecules	Class	Mechanism of action
[66]	P218	PfDHFR (Diaminooyridine)	Dihydrofolate reductase inhibitor
[67]	DSM265	Triazolopymrimidine	Dihydroorotate dehydrogenase
[68]	Decoquinate	PfCYTbc_1_	Cytochromebc1
[69]	KAF156	PfCARL	Cyclic amine resistance locus protein
[21]	21A092	Pyrazole	Unknown
[70]	ELQ-300	Quinolone-3-diarylether	Cytochrome *bc*1
[71]	RKA182	1,2,4,5-tetraoxane	Hemoglobin digestion
[72]	BCX4945	Immucillin G	Purine nucleoside phosphorylation
[73]	NPC-1161B	8-aminoquinoline	Unknown
[74]	SB939	PfHDAC1	Histone deacetylase
[75]	Falcitidin	PfFP2-3	Falcipain cysteine protease 2-3
[76]	GSK932121	PfCYTbc_1_	Cytochromebc1
[28]	SJ557733	PfATP4	Na + −ATPase 4
[77]	Trichostatin A	PfHDAC1	Histone deacetylation
[78]	TCMDC-134674	PfCHT1,2,4	Aspertic protease plasmepsins 1,2,4
[79]	E6446	TLR-9	Proinflammatory cytokines antagonist
[80]	MK4815	Aminoindoles	Mitochondrial electron transport chain inhibitor
[81]	Genz668764	Carboxamide	DHOD inhibition
[82]	RKA 182	1,2,4,5-tetraoxane	Hemoglobin digestion

## Synthetic medicinal arsenals

### OZ277 and OZ439

Quinine, first used in Europe in the 17th century, chloroquine [34], and 4-aminoquinoline scaffolds are some of the semi-synthetic drugs that have shown good antimalarial activity over the years. Fixed-dose combinations of artemisinin derivatives are currently considered to be the gold-standard malaria treatment. Synthetic artemisinin-like endoperoxides and their derivatives (artesunate, artemether, and dihydroartemisinin) have been proven to be more effective than chloroquine. OZ277 (arterolane), a novel non-artemisinin ozonide compound, has been developed by Ranbaxy Laboratories in collaboration with MMV in 2004. The clinical activity of OZ277 in a Phase II dose-finding trial for uncomplicated *P. falciparum* malaria was shown to be not as effective as artemisinin. This was indicated by the reduced parasitic clearance on day 28 after seven days (60–70 %) compared to artesunate dose–response (95 %) [34]. Thus, increasing the dose does not necessarily decrease parasitic recrudescence. Following a Phase III trial in 2013, a fixed-dose combination of OZ277 (arterolane) (150 mg) and long-acting piperaquine (750 mg) (Synriam™) was tested for treating *P. falciparum* malaria in India and received mark approval from the Drug Controller General of India. It was subsequently put on the market in seven African countries [35]. Due to a suboptimal half-life and reduced stability in low-level parasitemia (1 % at 45 % haematocrit), OZ277 failed to show efficacy against high parasitic loads [36]. The results of these trials confirmed the safety of the compounds but not the efficacy, even when doses were increased in the presence of a high level of infected erythrocytes. Drug partners may be needed to increase efficacy. These weaknesses have led MMV to develop a potential next generation synthetic endoperoxide ozonide, OZ439 (artefenomel), which has a longer half-life (30 h) and a MIC of more than one week, after a single dose. OZ439 is the first highly active ozonide against *Plasmodium* [36].

Different doses of artefenomel (200–1 200 mg) were tested in a Phase IIA exploratory, open-label trial and revealed promising safety and efficacy profiles among Southeast Asian adults with uncomplicated *P. falciparum* and *P. vivax* malaria. Due to the reduced elimination half-life of 46–62 h, a single dose of OZ439 alone or in combination with piperaquine can eliminate 98.0 % of *P. falciparum* and 99.6 % of *P. vivax* within 36 h. Artefenomel has demonstrated a higher parasitic clearance within the first 24 h in *P. vivax* patients as compared to *P. falciparum* patients (30–36 h). However, gametocyte clearance was 100 % in patients who were administered 1 200 mg of artefenomel within 48 h [36]. OZ439 is now being evaluated with piperaquine in Phase IIB combination trials.

One of the major concerns about the use of OZ compounds is that they have a similar endoperoxide structure to artemisinin, indicating possible treatment failures. Previous data suggest that artemisinin derivatives are associated with the risk of spontaneous abortions in early pregnancies [37], but recent clinical evidence confirmed the safety of ACTs against *P. falciparum* and *P. vivax* in the first trimester, with no risk of spontaneous abortions or major congenital malformations [38]. Similarly, preclinical studies have shown that OZ compounds are also safe for embryos and fetuses [39]. No clinical data are yet available to prove the safety of using these compounds in pregnancy and more tests are therefore needed for their evaluation.

### Other compounds

Two interesting endoperoxides from artesunate derivatives including artemisone (BAY 44–9585) and tetraxoane (TDD E209), are examples of other synthetic candidates currently under development. Artemisone is a semi-synthetic second-generation artemisinin derivative developed in collaboration between Bayer HealthCare Pharmaceuticals in Germany and the Hong Kong University of Science and Technology. Results of preclinical studies are highly promising as compared to other novel artemisinins. Artemisone is more effective than artesunate against *P. falciparum* and multidrug resistant strains [40, 41]. Dose-escalating Phase I trials on healthy volunteers have shown that artemisone is a rapidly effective treatment as it achieves peak plasma concentrations within 30 min following oral administration [41]. A Phase II interventional study testing artemisone for treating uncomplicated *P. falciparum* malaria planned for Western Cambodia (NCT00936767) has been withdrawn for unknown reasons. Some studies reported neurological and auditory side effects such as ataxia and slurred speech [42, 43] due to ACTs. However, no strong evidence exists to confirm neurological side effects. Furthermore, the activity of artemisone has shown a GM IC_50_s correlation with *pfmdr1* Y184F mutations, which potentially reduces sensitivity to artemisinin-resistant strains and contributes to emerging ACT resistance [44]. Recent genome-wide association studies revealed that artemisone does not interact with Y1915 and has no effect on *P.falciparum* phosphatidylinositol-3 kinase (PfPI3K) [45].

## Aminoquinoline scaffolds

### Ferroquine

Ferroquine is an ameliorated blood-schizonticidal 4-aminoquinoline developed by Sanofi-Aventis. Along with OZ439, it is a more effective parasite-killing compound against *Plasmodium* strains when compared to artesunate. Several preclinical studies have shown its benefits, particularly for treating patients infected with chloroquine-, amodiaquine-, and mefloquine-resistant malaria strains [46–48]. The greatest advantage of using ferroquine is its 30-h half-life, which is highly superior to that of other artemisinin derivatives. Two ferroquine Phase II trials were recently registered in the clinicaltrials.gov database (NCT02497612 and NCT00988507) focusing on *P. falciparum* and *P. vivax* malaria at the multicenter level. One study has been completed (NCT00988507) but no results are yet available. Most recently, a ferroquine-artesunate dose-ranging Phase II trial on *P. falciparum*-infected adults and children in eight African hospitals was conducted [49]. The research findings were astonishing: 97 % polymerase chain reaction (PCR)-confirmed cure rates (95 % *CI*: 90–100) after treatment with 2 mg/kg ferroquine combined with 4 mg/kg artesunate. However, the cure rate was reduced (79 %; 95%*CI*: 68–88) when ferroquine monotherapy 4 mg/kg/day for 3-days regimen was used. Furthermore, exacerbated malaria symptoms were observed in 14 % of the individuals in the treatment cohort.

### AQ-13

Another 4-aminoquinoline derivative called AQ-13 (Ro47-0543), a similarly structured chloroquine with a modified propyl side chain from the aminoquine panel, was developed in collaboration between Tulane University and Louisiana State University both located in LA, USA. Preclinical studies have indicated increased efficacy of AQ-13 when compared to other derivatives [50]. Phase I first-in-human safety and efficacy studies have shown results similar to those observed with chloroquine. Adverse events include electrocardiac changes, especially prolonged QTc intervals, which are commonly encountered with many quinolones [51]. AQ-13 did not present any advantages over other aminoquinolines, and further observation of this compound has currently been halted.

### Tafenoquine

The majority of clinical trials focus on malaria caused by *P. falciparum*, while fewer studies evaluate treatments against *P. vivax* and *P. ovale* malaria. A deficiency of glucose-6-phosphate dehydrogenase (G6PD) is a hereditary enzyme defect condition that causes episodic hemolysis. Patients with a G6PD deficiency are common in malaria-endemic countries and are at high risk of hemolysis due to treatment with antimalarial drugs (primaquine, chloroquine, quinine, and sulfamethoxazole). These patients are generally not included in trials due genotypic variations. For these individuals, tafenoquine (WR 238605) is a good alternative drug. It is an 8-aminoquinoline derivative and has a similar mode of action to primaquine against hypnozoites, gametocytes, and liver stages [52]. Tafenoquine is more potent during blood stages due its longer half-life (14 days) as compared to primaquine. Nevertheless, slower parasitic clearance was observed with tafenoquine monotherapy. Therefore, combing tafenoquine with other partner drugs may ideally benefit G6PD-deficient patients. So far, chloroquine combined with primaquine has been used for the radical cure of *P. vivax* malaria. Tafenoquine with chloroquine was tested in studies against *P. vivax* malaria. In a Phase IIB dose-ranging trial, different doses of tafenoquine alone (50, 100, 300, or 600 mg) or in combination with 15 mg primaquine for 14 days were tested, with a fixed dose of chloroquine for three days. A single dose of tafenoquine (300 mg) co-administered with chloroquine was shown to prevent relapse in 89.2 % (95 % *CI*: 77–95) of people as compared to chloroquine alone (51.7 %; 95 % *CI*: 36–69) during the first six months of follow-up [53]. Recent results of a Phase IIB dose-ranging trial (DETECTIVE study) conducted on monoinfected *P. vivax* patients for radical cure showed that single-dose tafenoquine (300 mg) combined to chloroquine is more efficacious in preventing relapses as compared to chloroquine alone, with a similar safety profile. Based on these observations, GSK and MMV announced two new Phase III studies: 1) a DETECTIVE study (TAF112582) to evaluate the efficacy, safety, and tolerability of tafenoquine co-administered with chloroquine as a radical cure for *P. vivax* malaria (blood-stage antimalarial treatment); and 2) a GATHER study (TAF 116546) to assess the incidence of hemolysis and the efficacy and safety of tafenoquine over primaquine [54].

## Biomolecular approaches

### Methylene blue

A century ago, the German scientist Paul Ehrlich discovered the antiplasmodial activity of methylene blue [55]. The chemotherapeutic use of synthetic methylene blue in treating methemoglobinemia and cancer-induced neurotoxicity was tested in 1995 [56]. Additional experiments were conducted using methylene blue and its analogs against *P. falciparum* isolates [57]. Methylene blue combined with chloroquine has been shown to prevent hemolysis in G6PD-deficient adult patients. Other studies assessed the use of different doses of methylene blue with chloroquine for three days and showed 90 % recovery rates in patients with uncomplicated *P. falciparum* malaria. Although results were promising, adverse effects were reported including vomiting, as well as the discoloration of urine, mucous surfaces, and teeth [58]. Drug resistance to chloroquine has also emerged globally [59]. In 2006, methylene blue was evaluated in combination with artesunate but showed poor cure rates despite rapid parasitic clearance [60]. In 2011, treatment with artesunate-amodiaquine-methylene blue was studied in children aged between six and 50 months with uncomplicated *P. falciparum* malaria*.* This combination showed poor efficacy (71 %) when compared to the control group (artesunate-amodiaquine; 85 %) [61]. However, after comparing a fixed dose 15 mg/kg of methylene blue co-administered with artesunate or amodiaquine versus artesunate-amodiaquine for three days, decreased gametocytes (from 100 to 36 %) were reported within seven days of treatment. Interestingly, the pronounced effect on gametocyte clearance indicates that methylene blue is a new promising drug component to reduce *P. falciparum* transmission. A Phase I trial testing the combination of methylene blue with primaquine is currently registered in the clinicaltrials.gov database (NCT01668433), but results are not yet available.

## Antibiotics

### Fosmidomycin

Isoprenoids are derived from the mevalonate pathway in humans, an essential metabolic pathway for parasite synthesis. Jomaa Pharma GmbH developed a synthetic antibiotic agent called fosmidomycin derived from *Streptomyces lavendulae* bacterial isolates. This compound inhibits the non-mevalonate pathway (also known as the DOXP pathway), essential for the synthesis of parasite isoprenoids [62]. Fosmidomycin has a half-life of only two hours and acts rapidly upon oral administration. Additional trials to evaluate the efficacy of different doses of fosmidomycin monotherapy undertaken for more than four days are required. One study indicated complete parasitic clearance on day seven following administration of fosmidomycin (1 200 mg four times a day) in adult patients with uncomplicated *P. falciparum* malaria. On day 28, recrudescence was observed in seven out of nine patients, indicating monotherapy failure [63]. Fosmidomycin co-administered with clindamycin has been proven to be effective in adults and older children with acute uncomplicated *P. falciparum* malaria. Poor efficacy was observed due to poor immunity in children aged between one an>d two years [64]. Two additional short half-life combinations (fosmidomycin with artesunate) were evaluated in 50 children aged between six and 12 years. Five different fosmidomycin-artesunate regimens achieved complete cure rates within three days of administration, and no resistant alleles were detected after seven and 28 days [65]. However, no evidence of prolonged protection by this combination was provided. A Phase IIA open-label efficacy trial focusing on fosmidomycin (450 mg capsule; twice daily) and piperaquine (320 mg; once daily) for treating patients with uncomplicated *P. falciparum* malaria, aged between one and 60 years and with a body weight between 5 and 90 kg, is currently registered in the clinicaltrials.gov database (NCT02198807). Overall, studies indicated that fosmidomycin is only effective for short-term treatment. Studies on finding a potential partner drug to prove the efficiency of fosmidomycin urgently need to be conducted.

## Conclusions

In this review, we summarized the different approaches tested over the years to control the malaria pandemic, and possibly reduce global malaria incidence and mortality by 90 % before 2030. Novel chemotherapeutic approaches have emerged over the past five years, with promising results. Nevertheless, the efficacy and safety of these drugs need to be studied further. These novel antimalarial approaches are multifaceted, thus there is an urgent need for effective single-dose molecules to act during the liver and blood stages of malaria. Effective compounds should be developed before global emergence of resistance to artemisinin derivatives and 4-aminoquinoline. There is currently no low-dose primaquine regimen for pediatric use. Novel blood-stage compounds such as DDD107498 and tafenoquine should focus on blocking parasite transmission in children and adolescents, and pregnant women. Molecules such as ferroquine should be combined with a potential partner drug to enhance efficacy. Additional challenges in preventing the relapse of malaria episodes include hemolysis in patients with a G6PD deficiency, treatment for drug-resistant strains, pediatric dosing, serious drug-drug interactions, transmission blocking, radical cure, and relapse prevention. Potentially targeting mitochondrial electron-transport chain of *P.falciparum* and protein inhibition in blood- and liver-stage parasites could be ideal for future drug development.

## Additional file

Additional file 1: Multilingual asbtracts in the five official working languages of the United Nations. (PDF 561 kb)

## Abbreviations

ACT: Artemisinin-based combination therapy
G6PD: Glucose-6-phosphate dehydrogenase
GMAP: Global Malaria Action Plan
GSK: GlaxoSmithKline
MIC: Minimum inhibitory concentration
MMV: Medicines for malaria venture
PfATP4: Na^+^-ATPase 4 ion channel
SSA: Sub-Saharan Africa
WHO: World Health Organization
