# Supplementary material for: Alternatives to currently used antimalarial drugs: in search of a magic bullet
Source: Infect Dis Poverty. 2016 Nov 4;5:103. doi: 10.1186/s40249-016-0196-8 (PMC5095999; doi:10.1186/s40249-016-0196-8)
Supplement: Additional file 1: — Multilingual asbtracts in the five official working languages of the United Nations. (PDF 561 kb) [file 40249_2016_196_MOESM1_ESM.pdf]

Translation of the abstract into the five official working languages of the United Nations

بدائل للادوية المضادة للملاريا المستخدمة حالياً: فى البحث عن الرصاصة السحرية

اكشايا سريكناث باجاڤاتولا، آسيم احمد النور، و عبد الله شهاب

خلاصة

الملاريا سبب رئيسى من أسباب المرض والوفاة فى بلاد أفريقية عديدة واجزاء من آسيا وأمريكا الجنوبية. سبل حديثة لمكافحة المرض ظهرت منذ سنوات قريبة وادوية عديدة مرشحة حاليا تحت الأختبار التجريبي. برغم هذا، سيطول وقت وصول هذه الأدوية الى الاسواق، هذا يرجع بخاصة الى نقص بيانات السلامة والفاعلية.

لتقليل عبأ الملاريا، مشروع أدوية الملاريا (MMV) انشا فى ١٩٩٩ لتطوير أدوية جديدة من خلال التعاون الصناعي والأكاديمي. مع ذلك، لم توجد مراجعات ممركزة بعد نشر دراسات تجريبية وابتدائية عديدة منذ مبادرت الMMV وحتى اليوم.

حددنا مناهج واعدة فى الملف العالمي للادوية المضادة للملاريا، وأوضحنا التحديات وأسباب محدده لقلق المريض من هذة الجزيئات الجديدة. ناقشنا أبحاث تجريبية مختلفة فى تقدير الأدوية الجديدة ضد الملاريا فى تجارب بشرية خلال الخمس سنوات الماضية.

الدواءين 609KAE و 107498DDD مازالوا تحت التقييم فى الجزء الاول من التجربة و فى دراسات التطوير الابتدائية. السلامة والفاعلية كلاهما فى تركيبة جديدة مثل 156KAF و 265DSM يحتاجون مزيد من التقييم، بخاصة فى إمكانية استخدامهم لعلاج الحوامل. التركيبات الصناعية غير ارتيميسينين أوزونيد أثاروا قلق تجاه قلت كفاءتهم ضد التحميلات الطفيلية. بنيات مصدرها الامينوكينولين مثل فروكوين واعدين ولكن يجب دمجهم مع أدوية شريكة جيدة لزيادة الفاعلية. 13-AQ أدى الى تواجد تغيرات فى كهربة القلب مما أدي الى تطويل مسافة QTc. تافنكوين، هو البنية الوحيد ضد عودة المرض للمرضى الذي لديهم نقص فى جلوكوز-٦-فوسفات دهيدروجناز، أدى الى ظهور عدة مشاغل مهمة ذلك لنشاطة الهيمولتك. مكونات اخري، منها المثلين الأزرق (حاجز توصيل محتمل) و فوزميدوميسين (DXP ردكتوازومراز مانع)، متوفرين لكن لا يمكن استخدامهم للاطفال.

فى هذة المرحلة، لا يمكننا تحديد رصاصة سحرية واحدة ضد الملاريا. دراسات مستقبلية يجب ان تركز على جرعة واحدة لجزئيات فعالة ويمكنها العمل ضد جميع مراحل الملاريا لمنع الانتقال. أدوية احدث اثارت شك من ناحية الفاعلية والسلامة. بوجهاً عام، باحتياج المزيد من الأدلة لتقليل عبأ الملاريا الحالى. استراتجيات علاج تستهدف مرحلة الدم مع خواص منع-النقل مطلوبة لمنع مقاومة العقاقير.

Translated from English version into Arabic by Chouvac, through

TRANSLATORS
WITHOUT BORDERS

寻找替代现有抗疟药物的灵丹妙药

Akshaya Srikanth Bhagavathula, Asim Ahmed Elnour, **Abdulla Shehab**

摘要

疟疾是许多非洲国家、亚洲和南美部分地区发病和死亡的重要病因。近年来出现了许多新颖的抗疟措施，并且一些候选药物正处于临床试验中。然而，这些新药入市还需要很长时间，特别是还缺少安全性和有效性的相关数据。

为减轻疟疾疾病负担，于 1999 年成立疟疾药物开发项目（Medicines for Malaria Venture，MMV），旨在通过工业界与学术界合作研发抗疟新药。然而项目成立至今，尚无针对已发表的各种临床前和临床研究的系统综述。我们确定了全球一些有前景的抗疟药，并突出了这

些新药所面临的挑战和患者特别关注的内容，且对过去 5 年中在不同人群中评价抗疟新药的不同临床研究进行了讨论。

药物KAE609和DDD107498仍处于I期试验阶段和临床前研发中。像KAF156和DSM265这样的新化合物，其安全性和有效性有待进一步评估，尤其是应用于孕妇。例如，OZ277的合成型非青蒿素臭氧化物在治疗高原虫血症时缺乏有效性的资料已引起关注；二茂铁氯喹这种基于氨基喹啉类药物具有很好的前景，但为了进一步提高有效性，需要与好的配伍药联合用药。AQ-13 诱导心电活动，导致 QTc 间隔延长。Tafenoquine 是唯一针对 G-6-PD 缺乏患者的抗复发新化合物，而由于其溶血作用引起重大关注。还有其他化合物，包括亚甲基蓝（潜在的传播阻断剂）和膦胺霉素（还原异构酶阻滞剂）可用，但不能用于儿童。

目前这个阶段，尚未发现单一有效的抗疟灵丹妙药。未来应重点关注那些在疟原虫所有发育阶段均能发挥有效作用从而阻断传播的单次给药的分子。同时对新药的安全性和有效性也应重视。总之，还需要更多的证据来有效减轻当前疟疾疾病负担。需要靶向血期疟原虫兼具传播阻断特性的治疗策略来预防今后的药物抗性。

Translated from English version into Chinese by Jian-Hai Yin, edited by Pin Yang

CHINESE CENTER FOR DISEASE CONTROL AND PREVENTION
NATIONAL INSTITUTE OF PARASITIC DISEASES

Alternatives aux antipaludéens utilisés actuellement : à la recherche de la formule magique

Akshaya Srikanth Bhagavathula, Asim Ahmed Elnour, et Abdulla Shehab

Résumé

Le paludisme est une cause majeure de morbidité et de mortalité dans de nombreux pays d'Afrique, d'Asie et d'Amérique du Sud. De nouvelles stratégies de lutte contre la maladie sont apparues ces dernières années et plusieurs médicaments candidats sont en cours d'essais cliniques. Il faudra cependant longtemps pour que ces nouvelles molécules arrivent sur le marché, à cause notamment du manque de données d'innocuité et d'efficacité.

Afin de réduire le poids du paludisme, Medicines for Malaria Venture (MMV) a été créé en 1999 dans le but de développer de nouveaux médicaments dans le cadre d'une collaboration entre l'industrie et les universités. Les différents essais précliniques et cliniques publiés depuis la création de MMV n'ont cependant pas encore fait l'objet d'une revue à ce jour.

Nous identifions des approches prometteuses dans le portefeuille global de médicaments antipaludéens et mettons en évidence les difficultés de leur développement et les préoccupations concernant les patients avec ces nouvelles molécules. Nous discutons de différents essais cliniques menés ces cinq dernières années sur des sujets humains pour évaluer de nouveaux médicaments contre le paludisme.

Les molécules KAE609 et DDD107498 sont encore en cours d'évaluation dans le cadre d'essais de Phase I et d'études de développement préclinique. L'innocuité et l'efficacité des nouveaux composés tels que KAF156 et DSM265 doivent encore être évaluées, en particulier leurs effets pendant la grossesse. Les ozonides de synthèse non artémisinine tels qu'OZ277 ne semblent pas suffisamment efficaces lorsque la charge parasitaire est importante. Les aminoquinolines telles que la ferroquine sont prometteuses mais doivent être associées judicieusement à d'autres molécules

afin de renforcer leur efficacité. AQ-13 est associé à des événements électrocardiaques, avec allongement de l'intervalle QTc. La tafénoquine, seul composé empêchant les rechutes chez les patients présentant un déficit en glucose-6-phosphate déshydrogénase, présente une inquiétante activité hémolytique. D'autres composés, comme le bleu de méthylène (qui pourrait bloquer la transmission) et la fosmidomycine (inhibiteur de la DXP réducto-isomérase), sont disponibles sur le marché mais ne peuvent pas être utilisés chez l'enfant.

Dans l'état actuel des données, nous n'avons pas pu identifier un composé unique qui serait notre « formule magique » contre le paludisme. Les études futures devront se concentrer sur les molécules efficaces en dose unique, capables d'agir sur tous les stades du paludisme, afin d'empêcher la transmission. Les nouveaux médicaments posent aussi des problèmes d'efficacité et d'innocuité. Dans l'ensemble, il faudra davantage de données pour savoir comment réduire efficacement le fardeau actuel du paludisme. Nous avons besoin de stratégies de traitement visant le stade sanguin et bloquant la transmission afin d'empêcher le développement futur de résistances.

Translated from English version into French by Suzanne Assenat, through

TRANSLATORS
WITHOUT BORDERS

Альтернативы используемым в настоящее время противомалярийным препаратам: в поисках чудодейственного средства

Акшая Шрикант Бхагаватула, Азим Ахмед Эльнур и Абдулла Шехаб

РЕФЕРАТ

Малярия является основной причиной заболеваемости и смертности во многих странах Африки, Азии и Южной Америки. В последние годы в борьбе с этой болезнью сделаны инновационные открытия, и в настоящее время проводятся клинические испытания нескольких потенциальных лекарственных препаратов. Однако, пройдет немало времени прежде чем новые лекарственные препараты смогут поступить в продажу, особенно вследствие ограниченности данных по их безопасности и эффективности.

Для борьбы с малярией в 1999 году было создано предприятие «Лекарства против малярии» (MMV), ставящее перед собой задачу разработки новых лекарственных препаратов путем организации сотрудничества между промышленностью и академическими кругами. Однако, по результатам предклинических и клинических исследований, опубликованных с начала деятельности MMV (2000 г.) по нынешний день, не было сделано никаких критических обзоров.

Нами отмечаются многообещающие открытия в сфере противомалярийных препаратов и выделяются задачи и относящиеся к пациентам проблемы, связанные с этими новыми препаратами. Обсуждаются различные клинические исследования с заострением внимания на оценке новых противомалярийных лекарственных средств в клинических исследованиях с участием людей, проведенных за последние пять лет.

Лекарственные препараты KAE609 и DDD107498 все еще проходят оценку на первой стадии клинических исследований и предклинических экспериментальных исследований. Требуют дополнительной проверки как безопасность, так и эффективность новых препаратов, как, например, KAF156 и DSM265, особенно для применения у беременных женщин. Такие синтетические озониды, не содержащие артемизинина, как OZ277 вызывают обеспокоенность в связи с их недостаточной эффективностью в отношении паразитных нагрузок. Производные аминохинолина, как, например, феррокин (ferroquine), обладают большим потенциалом, но для повышения их эффективности необходимо использовать их в сочетании с хорошими сопутствующими лекарственными средствами. AQ-13 приводит к возникновению коронарных событий, вызывающих удлиненные интервалы QT. Тафенохин (Tafenoquine), единственный новый противорецидивный препарат для пациентов с дефицитом глюкозо-6-фосфатдегидрогеназы, вызвал серьезную озабоченность вследствие его гемолитического воздействия. Другие лекарственные препараты, включая метиленовый синий (methylene blue) (потенциальный антитрансмиссионный блокатор) и фосмидомицин (fosmidomycin) (ингибитор энзима DXP reductoisomerase) выпущены к использованию, но не могут применяться при лечении детей.

На этой стадии не существует ни одного чудодейственного противомалярийного средства. Будущие исследования должны быть сосредоточены на эффективных препаратах с однократным приемом, действующих на всех стадиях малярии для предупреждения ее передачи. Новые препараты вызвали озабоченность также в связи с их действенностью и безопасностью. В целом требуется больше данных для эффективного сокращения бремени малярии. Для предупреждения возникновения устойчивости к лекарственным препаратам необходимы стратегии лечения, направленные на развитие антитрансмиссионных свойств на стадии распространения малярии в кровеносном русле.

Translated from English version into Russian by Alena Hrybouskaya, through

TRANSLATORS
WITHOUT BORDERS

Alternativas a los fármacos antimaláricos actuales: en búsqueda de una fórmula mágica

Akshaya Srikanth Bhagavathula, Asim Ahmed Elnour y Abdulla Shehab

RESUMEN

La malaria es una causa importante de morbilidad y mortalidad en muchos países africanos y algunas partes de Asia y de Sudamérica. En los últimos años, han surgido enfoques innovadores para combatir la enfermedad y actualmente se están probando clínicamente varios fármacos candidatos. Sin embargo, todavía falta mucho tiempo hasta que estos fármacos novedosos puedan llegar al mercado, debido, especialmente, a la escasez de datos de seguridad y de eficacia.

Para reducir la carga de la malaria, en 1999, se fundó la *Medicines for Malaria Venture* (MMV) con el objetivo de desarrollar medicamentos novedosos a través de una colaboración entre la

industria y socios del ámbito académico. No obstante, ninguna revisión se ha centrado en el seguimiento de los diversos estudios preclínicos y clínicos publicados desde el inicio de la MMV en el año 2000 hasta la fecha.

Identificamos enfoques prometedores en la cartera de productos global de medicamentos antimaláricos y apuntamos desafíos y preocupaciones específicas de los pacientes en relación a estas innovadoras moléculas. Debatimos diferentes estudios clínicos enfocados en la evaluación de fármacos innovadores para combatir la malaria en diferentes ensayos en humanos a lo largo de los cinco últimos años.

Los fármacos KAE609 y DDD107498 todavía están siendo evaluados en ensayos de Fase I y estudios de desarrollo preclínico. Es necesario evaluar más a fondo tanto la seguridad como la eficacia de los nuevos compuestos, como KAF156 y DSM265, especialmente su uso en mujeres embarazadas. Surgió una preocupación por la eficacia insuficiente de los ozónidos sintéticos sin artemisina como OZ277 frente a las cargas parasitarias altas. Los fármacos basados en la aminoquinolina, como la ferroquina, son prometedores, pero para realzar su eficacia se deben combinar con otros medicamentos. El AQ-13 indujo acontecimientos electrocardíacos cuya consecuencia fue la prolongación del intervalo QTc. La tafenoquina, el único medicamento nuevo para combatir las recidivas en pacientes con déficit de glucosa-6-fosfato deshidrogenasa, ha planteado problemas significativos debido a su actividad hemolítica. Están disponibles otros compuestos, tales como el azul de metileno (posible bloqueante de la transmisión) y la fosmidomicina (inhibidor de la reductoisomerasa DXP), pero no se pueden emplear en niños.

En este momento, no podemos identificar una sola fórmula mágica para combatir la malaria. Los estudios futuros deberían estar centrados en moléculas de dosis única eficaces y capaces de actuar en todas las etapas de la malaria con el fin de prevenir su transmisión. Los medicamentos más novedosos también han planteado problemas de eficacia y seguridad. En resumen, son necesarias más evidencias para poder reducir eficazmente la carga actual de la malaria. Para prevenir futuras resistencias a fármacos, son necesarias estrategias terapéuticas dirigidas a la etapa hemática y capaces de bloquear la transmisión.

Translated from English version into Spanish by Sión Guitart, through

TRANSLATORS
WITHOUT BORDERS
